# Is It Words or Things?

**Published:** 1902-09

**Authors:** 


### Is It Words or Things?

OUR highly esteemed contemporary the *Cleveland Medical Journal*, always positive, generally accurate, and often right, in its issue for August, reports the interesting fact that we have taken away its breath with our recent statement that "There are no sects in medicine known to the law" and courteously suggests that we further elucidate the subject. This we hasten to undertake, but first pause for an instant to record the hope that the suspension of respiration upon the part of our esteemed friend was quite transitory, and that no sub-oxidation, cerebral or otherwise, resulted therefrom, remembering that shortage in air supply is frequently inconvenient, and generally disagreeable; and further that normal cerebration is essential to the right understanding of this important topic even after full elucidation.

Our title suggests and to many will make the complete elucidation. In brief the answer is, that to those content to be satisfied with the search for words rather than the facts which words simplify, our statement as to the law and the sects is quite inaccurate, misleading, or false as one will; to those who are accustomed to see through words to things, our language although possibly condensed is yet substantially accurate.

By the "law" we do not mean the words of the statute or the decree of the court interpreting that statute, but we do mean the condition of the medical profession resulting from the law, its relation to the people, to vital statistics to life insurance, to the trial of medico-legal cases, to expert testimony before the courts, to medical services, to examinations of candidates for license to practise medicine, to voting upon the fitness of candidates for such license, and in fact upon every act a doctor may do or not do, of which the law of the land may take cognisance; and in each and all of these instances the law,—that is to say, the condition produced by the law, the condition intended by the statute and the decree of the court interpreting that statute to be the result of the law in such cases made and provided,—knows no sects in medicine.

When our highly esteemed contemporary argues to the contrary of this statement he undertakes to prove that those who think, and are rather proud to think that they belong to the medical profession, are sadly mistaken; that in fact they are only members of the older and more numerous of the medical sects.

